# Development and evaluation of an automated phase contrast magnetic resonance imaging algorithm for pediatric and adult cerebral blood flow measurement

**DOI:** 10.1007/s00234-026-03948-3

**Published:** 2026-03-27

**Authors:** Joseph Liu, Isabel Torres, Sudarshan Ranganathan, Bethany L. Sussman, Eamon K. Doyle, Abhishek Karnwal, Samantha T. Nimmo, Benita Tamrazi, Bradley J. De Souza, Peter A. Chiarelli, Meredith N. Braskie, Hussein N. Yassine, John C. Wood, Bradley S. Peterson, Matthew T. Borzage

**Affiliations:** 1 Fetal and Neonatal Institute, Division of Neonatology, Children’s Hospital Los Angeles, Los Angeles, USA; 2 Department of Radiology, Children’s Hospital Los Angeles, Los Angeles, USA; 3 Rudi Schulte Research Institute, Santa Barbara, USA; 4 Department of Anesthesiology Critical Care Medicine, Children’s Hospital Los Angeles, Los Angeles, USA; 5 Division of Cardiology, Department of Pediatrics, Children’s Hospital Los Angeles, Los Angeles, USA; 6 Mark and Mary Stevens Neuroimaging and Informatics Institute, USC Keck School of Medicine, Los Angeles, USA; 7 Division of Neurosurgery, Children’s Hospital Los Angeles, Los Angeles, USA; 8 Center for Personalized Brain Health, USC Keck School of Medicine, Los Angeles, USA; 9 Institute for the Developing Mind, Children’s Hospital Los Angeles, Los Angeles, USA; 10 Department of Psychiatry, USC Keck School of Medicine, Los Angeles, USA; 11 Alfred E. Mann Department of Biomedical Engineering, University of Southern California Viterbi School of Engineering, Los Angeles, USA; 12 Department of Regulatory and Quality Sciences, Alfred E. Mann School of Pharmacy and Pharmaceutical Sciences, University of Southern California, Los Angeles, USA

**Keywords:** Cerebral blood flow, Hemodynamics, Magnetic resonance imaging

## Abstract

**Purpose:**

Accurate Cerebral Blood Flow (CBF) measurements are essential for studying pediatric cerebral hemodynamics. Phase Contrast (PC) imaging is a fast, non-invasive, and non-radiating technique for measuring flows. PC image processing traditionally includes manually segmenting, identifying, and unaliasing vessels of interest, which are challenging in children and involve intra- and inter-observer variation.

**Methods:**

We acquired 3 T PC images from 59 children and 39 adults (mean and standard deviation 3.43 ± 2.60 and 57.28 ± 3.87 years). Our algorithm identified voxels that skew the PC image intensity, refined vessels with active contours (Chan-Vese), split adjacent vessels with watershedding, and used a heuristic to identify the correct arteries based on vessel characteristics. We developed an automated algorithm to process PC images, thereby ensuring high precision. Images were processed images manually (two analysts) and algorithmically to compare performance overall and for each component.

**Results:**

Total CBF measurements were correlated between ground truth and algorithm versus between two analysts (Intraclass Correlation Coefficient = 0.748—0.817 vs 0.810—0.919). A two-way analysis of variance indicated no difference between human and algorithm for total CBF (p = 0.1558). The performance of algorithm versus two human analysts were similar across components: segmentation (Matthew’s Correlation Coefficient = 0.777—0.849 vs 0.830—0.890), unaliasing (Mean Absolute Error = 0.355—0.538 vs 0.410—0.555), and vessel identification in adults (Intraclass Correlation Coefficient = 1.000 vs 1.000). Analysts were similar versus algorithm at vessel identification in children (Intraclass Correlation Coefficient = 1.000 vs 0.983).

**Conclusion:**

Automated algorithm components performed similarly to gold standard manual analysis and ensured high precision.

## Introduction

Cerebral Blood Flow (CBF) supplies the brain with oxygen and glucose, which are essential for healthy brain development and function [1]. Pediatric CBF measurements provide data for studying tumor diagnoses and treatment [2–4], disruptions in normal brain development [5, 6], vascular disease [6–8], and cognitive impairment [9]. Regional CBF measurements are important methods to differentiate between high- versus low-grade brain tumors [3, 4]. However, many regional CBF measurements are time-consuming, include imprecision from different image acquisition and processing methods, are invasive, or involve radiation. A fast, precise, noninvasive global measurement of total CBF would complement regional CBF measurements. We can obtain the ratio of total CBF from the regional versus global CBF measurements, and use that ratio to scale regional tumor perfusion measurements, thereby improving measurement accuracy. Thus, quick and precise measurements of global CBF would enhance studies of pediatric tumors and other pathologies.

Positron Emission Tomography (PET) is one method for measuring regional CBF accurately, requiring the injection and monitoring of radioactive ^15^O-labeled water as a radiotracer [10]. A newer method for regional CBF measurement is Arterial Spin Labeling (ASL), which uses Magnetic Resonance (MR) properties of blood as a noninvasive tracer. ASL requires relatively long imaging times, is prone to motion artifacts in the pediatric population, and its data processing often requires assumptions about cerebral hemodynamics [11]. Phase Contrast (PC) MR imaging is a noninvasive, radiation-free method for measuring global CBF [12]. Each PC image shows neuroanatomical structure, vascular anatomy, and blood velocity in the magnitude, complex difference, and phase components, respectively. Computing blood flow in a vessel requires selecting voxels in the vessel lumen, multiplying blood velocity in each voxel by the voxel cross-sectional area, and adding the values of all selected voxels (Eq. 1). In each PC image, total CBF is the sum of the flows in the 2 Internal Carotid Arteries (ICAs) and 2 Vertebral Arteries (VAs) (Eq. 2).

1 $${Flow}_{j}=\sum_{i=1}^{I}Velocity \times {Area}_{i,j}$$

2 $$\text{Total CBF}=\sum_{j=1}^{J}{Flow}_{j}$$

$$\begin{array}{c}i=\text{index of voxels in vessel}\\ I=\text{number of voxels in vessel}\\ \begin{array}{c}j=\text{index of vessel}\\ J=\text{number of vessels} (2\ \mathrm{ICAs} + 2\ \mathrm{VAs})\end{array}\end{array}$$

The challenges of acquiring high-quality PC images are well known, and there are recommended imaging approaches to address them [13, 14]. The PC imaging slice must be perpendicular to both ICAs and VAs, and images must have sufficient spatial resolution to visualize lumens of the 4 vessels [13, 14]. The Velocity Encoding (VENC) parameter, which dictates the sampled range of velocity and intensity values, must be as low as possible to maximize the signal to noise ratio, yet higher than the velocities in the vessel to avoid aliasing the phase image [12, 13, 15]. Selecting the correct VENC is challenging due to large velocity changes from birth to adulthood (18 ± 5 to 45 ± 15 cm/s mean and standard deviation) [16, 17]. Aliasing can be prevented by increasing the acquisition VENC or corrected in post-processing by phase-unwrapping the image. Although manually processing PC images is the gold standard, the major remaining challenge is reducing PC measurement error through systematic vessel identification, vessel segmentation, and aliasing correction.

Automated PC image processing to address segmentation and other issues during processing has the potential to eliminate inter- and intra-observer variation through precise global CBF measurements, and reduce manual processing time, thereby yielding information that complements regional CBF measurements [18]. An automated algorithm will have exact precision, and quantifying its performance across the lifespan will help determine its applicability in childhood during which there is a large variation in CBF. Performance limits are expected to be highlighted when measuring CBF in younger participants, as children have smaller vessels, lower flows, and tend to move during the MRI. Therefore, we report analysis of performance of our automated algorithm for PC processing in both a group of relatively easy-to-assess adult volunteers, and in a group of very difficult-to-assess pediatric patients.

## Materials and methods

### Participants

We acquired PC and T1 MR images for secondary analysis from 2 studies (one pediatric and one adult). In the pediatric study, PC imaging was added to routine neuroimaging for consented children (inpatients and outpatients) imaged at a quaternary pediatric academic hospital (IRB CHLA-18-00439, NCT04435834). Each PC imaging sequence added approximately 1 min to clinical scan time. Pediatric participants included those both with and without central nervous system pathologies such as tumors, neurological disorders, and other physiological conditions. In the adult study, participants were consented to receive research scans at an academic hospital (IRB CHLA-18-00439). Each PC imaging sequence took approximately 1 min. Adult participants included those who were non-diabetic, pre-diabetic, or diabetic.

Due to clinical time constraints, both pediatric and adult images were only acquired at a single VENC. However, evaluation of unaliasing required image pairs acquired at both low VENC (with aliasing) and high VENC (ground truth without aliasing). Thus, one consented adult (29 years) for whom we had time to collect image pairs was also scanned (IRB HS-22-00468). Pairs of PC images were acquired at low and high VENC (30 and 60 cm/s), thereby creating images with and without aliasing. PC imaging parameters and slice location for these adult images were equivalent to those in pediatric images.

### Data acquisition

All pediatric images were acquired on 3 T Philips (Amsterdam, Netherlands) Achieva, Achieva CX, and Ingenia MR scanners with software revision R5.7.1.2. The VENC, number of signals averaged, and isotropic in-plane resolution parameters were optimized for pediatric vessel sizes and velocities to reduce aliasing and increase contrast and signal to noise ratios. Key sequence parameters were: VENC 30—60 cm/s, echo time 7.41—17.32 ms, repetition time 12.20—27.17 ms, number of signals averaged 6—36, no cardiac gating, isotropic in-plane resolution 0.50—0.89 mm, slice thickness 5.00 mm, and Compressed SENSitivity Encoding (CSENSE) factor 2.

All adult images were acquired on a 3 T Siemens (Erlangen, Germany) Prisma scanner. The scanning parameters were optimized for adult vessel sizes and velocities. Key sequence parameters were: VENC 60 cm/s, echo time 4.89 ms, repetition time 47.34 ms, number of signals averaged 32, no cardiac gating, isotropic in-plane resolution 0.40 mm, slice thickness 5.00 mm, and GeneRalized Autocalibrating Partially Parallel Acquisitions (GRAPPA) factor 2.

For pediatric and adult PC images, we saved magnitude, complex difference, and phase information. The PC imaging slice was placed 1 cm above the carotid bifurcation, perpendicular to the bilateral ICAs and VAs. Pediatric images were acquired during a research addition to a clinical MR imaging. Because of this, the sequence was highly time-constrained and lead to lower resolutions and fewer averaged signals. In contrast, the adult images were acquired during a 2-h long neuroimaging protocol, which was far less time constrained.

### Brain volume image processing

For pediatric and adult T1 images, we processed T1 images using Free Surfer (7.2.0 for pediatric images, 5.3.0 for adult images, Laboratory for Computational Neuroimaging, Boston MA). If brain volumes were unable to be processed with Free Surfer due to unusual brain anatomy, we either used Computational Anatomy Toolbox (CAT12) (12.9, University of Jena, Germany) with Statistical Parametric Mapping (SPM12) and MATLAB (2023A MathWorks, Natick MA), or imputed their brain volumes based on their sex and age [19].

### Cerebral blood flow image processing

**Fig. 1:**
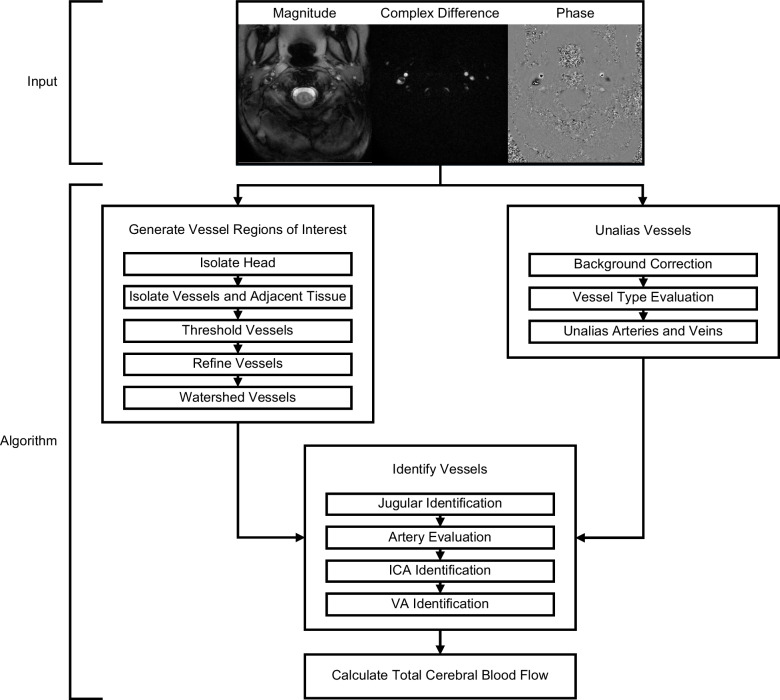
Our algorithm consists of 4 main components: generating vessel regions of interest, unaliasing vessels, identifying vessels, and calculating total cerebral blood flow. After the algorithm is complete, other steps such as normalizing by brain volume can be applied as desired. Abbreviations: Internal Carotid Artery (ICA), Vertebral Artery (VA)

Our PC processing algorithm was written in MATLAB (2023A MathWorks, Natick MA) and contains 4 components **(**Fig. 1**)**. These key components are: 1. vessel segmentation of ICAs, VAs, and internal jugular veins from the complex difference image, 2. vessel unaliasing of voxels in the phase image, 3. vessel identification of ICAs and VAs using both phase and complex difference images, and 4. CBF calculation using segmented and identified vessels, and the unaliased phase image.

## Vessel segmentation

The algorithm begins by segmenting the head from surrounding air and then segmenting vessels from tissues of the head.

### Isolate head

An active contour method (Chan-Vese) [20] processes the magnitude image to isolate head from surrounding air. The active contour begins with the entire image selected and iteratively (N = 200) shrinks the region to segment the cranial region of interest (ROI).

### Isolate vessels and adjacent tissue

An iterated histogram method processes the complex difference image to select voxels inside of the vessels and a region of surrounding tissue. This method identifies the voxel with the highest intensity within the segmented cranial ROI and creates a vascular ROI based on a small circle (radius 15 voxels) centered on that voxel. These vascular ROIs includes voxels that skew the data in the cranial ROI, hence they are excluded from the next iteration, allowing for the capture of both large and small regions that may be potential vessels. The process repeats until unselected voxels in the cranial ROI are no longer skewed (skewness < 0.8) or until the completion of 10 iterations.

### Threshold vessels

A threshold value (median amplitude of the unselected voxels plus 2.5 times the interquartile range of these voxels) is used to refine the vessel ROIs selected in the previous step. Voxels below this threshold (which may be static tissue or slowly moving cerebrospinal fluid) are removed from the vessel ROIs.

### Refine vessels

An active contour method (Chan-Vese) [20] refines the resulting vessel ROIs. The active contour method performs 3 iterations with a contraction bias, then dilates by 1 voxel to counterbalance the bias.

### Split (watershed) adjacent vessels erroneously marked as a single vessel

The ICA is in close proximity to the internal jugular vein, which can cause both vessels to be selected erroneously as a single ROI having both positive and negative flow. To prevent this, we perform a preliminary unaliasing step for the phase image (Ghiglia-Romero) [21] and watershed those ROIs into separate regions of positive and negative flows.

## Vessel unaliasing

Next, the algorithm evaluates if an aliased vessel is an artery or vein and then unaliases the vessel accordingly.

### Background correction

We correct for background phase by dilating our vessel regions by 1 voxel and applying the MATLAB regionfill function. Regionfill computes the Laplacian inside these regions while maintaining Dirichlet boundary conditions at the edges of the regions of interest. Then we subtract the regionfill image from the phase image, thereby removing any nonzero background phase within the vessels.

### Vessel type evaluation

Aliased vessels are evaluated using 4 parameters to determine if they are arterial or venous. The parameters are: A. ratio of voxels with positive (arterial) versus negative (venous) flow values, B. total vessel flow before unaliasing (Eq. 1), C. total vessel flow after preliminary unaliasing (Ghiglia-Romero) [21], and D. solutions for a parabolic flow profile fit to the vessel. Parameters A through D are assigned weights that provided the best results, and if the weighted sum of parameters exceeds 2, then the vessel is classified as an artery (Eq. 3).

$$\begin{array}{c}A=\left\{\begin{array}{c} 1, \text{if number of positive Velocity}_{i}>\text{number of negative Velocity}_{i}\\ 0, \mathrm{else}\end{array}\right.\\ B=\left\{\begin{array}{c} 2, \text{if Velocity}_{i,j} \times \mathrm{Area}_{i,j}>0\\ 0, \mathrm{else}\end{array}\right.\\ \begin{array}{c}C=\left\{\begin{array}{c} 1, \text{if Unaliased Velocity}_{i,j} \times \mathrm{Area}_{i,j}>0\\ 0, \mathrm{else}\end{array}\right.\\ D=\left\{\begin{array}{c} 1, \text{if best parabolic fit is positive and real}\\ 0, \mathrm{else}\end{array}\right.\end{array}\end{array}$$

3 $$vessel=\left\{\begin{array}{c} artery, \text{if A+B+C+D>2} \\ vein, \mathrm{else}\end{array}\right.$$

Phase aliasing corrupts the blood flow profile in vessels of interest. The expected flow profile in the ICA is a blunted parabola (Womersley number 5.33) [22], yet we picked a parsimonious parabolic model as a suitable simplification. Aliased vessels contain both positive and negative flow values within the same ROI, complicating their identification as an artery or vein. Our algorithm accounts for this ambiguity by unaliasing each aliased vessel as an artery and then a vein. Both possibilities are then fit to a parabolic profile. We reject impossible parabolic fits (those with imaginary solutions), select the fit with the best r-squared value, and use that fit to designate the weighting parameter D.

### Unalias arteries and veins

The algorithm designates a vessel as an artery if the total weight of the above classification parameters is greater than 2, otherwise the algorithm designates the vessel as a vein. Aliased voxels within the designated vessel lumen are then unaliased using the selected parabolic flow function.

## Vessel identification

The algorithm identifies the 2 ICAs and 2 VAs using the vessel ROIs and flows calculated previously.

### Internal jugular identification

The internal jugular veins are identified as the 2 vessel ROIs with the largest negative flows on the left and right sides of image midline.

### Artery evaluation

We first identify the vessel with the highest flow. All vessels with at least 20% of the highest flow are potential ICA candidates. Each of these candidates is evaluated on 4 measures to determine if it is an ICA (Eq. 4): A. distance to the anterior edge of image scaled by anterior–posterior image size, B. distance to the midline scaled by left–right image size, C. distance to closest internal jugular vein scaled by the sum of every ICA candidate’s distance to their closest internal jugular vein, D. vessel flow scaled by sum of all potential ICA flows. The ICAs are anatomically more anterior, lateral, and proximal to internal jugular veins compared with VAs; ICAs have higher vessel flows compared to VAs. We sum the 4 measures A, B, C, and D to get the total weight for each vessel.

$$\begin{array}{c}A=\frac{\text{Vessel Centroid Distance to Anterior Edge of Image}}{\text{Anterior to Posterior Image Size}}\\ B=\frac{\text{Vessel Centroid Distance to Midline of Image}}{\text{Left to Right Image Size}}\\ \begin{array}{c}C=\frac{\text{Vessel Centroid to Closest Jugular Vein Centroid Distance}}{\sum \text{Vessel Centroid to Closest Jugular Vein Centroid Distances}}\\ D=\frac{\text{Vessel Flow}}{\sum \text{Vessel Flows}}\end{array}\end{array}$$

4 $$\text{Total Weight}=A+B+C+D$$

### ICA Identification

The vessels with the largest total weighting on the left and right sides of the midline are identified as the bilateral ICAs.

### VA Identification

The algorithm uses the ICAs as references and identifies the VAs as the vessel with the next-highest flow within a specified distance (anterior: 10 voxels, lateral: 20 voxels) from the ipsilateral ICA.

## CBF Calculation

The algorithm then calculates total CBF using identified ICA and VA vessel ROIs and unaliased phase data. For each ICA and VA ROI, the flow velocity of each voxel from the phase image is multiplied by in-plane voxel area and summed to produce a vessel flow (Eq. 1). Total CBF is then calculated as the total flow summed from the 2 ICAs and 2 VAs, which completes the algorithm (Eq. 2).

### CBF Normalization

We note that many studies use ASL and PET, and thus they report normalized CBF value, i.e. units of ml/min/100 g [12]. Our algorithm does not require this normalization, but if normalized CBF is desired, this can easily be computed. Brain volume is available from other neuroimaging (e.g. T1 weighted images), and brain weight can be obtained by multiplying a reference scalar density (1.081 g/cm^3^) [23].

### Manual processing & comparison to algorithm

The primary and secondary image analysts analyzed all images manually in MATLAB (vessel segmentation, vessel identification, CBF calculation) and ImageJ (vessel unaliasing). Both analysts were blinded to algorithmic results. We performed statistical analyses to assess potential age-dependent differences in performance on pediatric and adult images. The primary image analyst performed manual analyses twice and results from the first observation were used as ground truth for vessel segmentation, identification, and CBF calculation. We selected the first assessment by our primary image analyst as our ground truth because it was the unbiased initial assessment of our most skilled analyst. Thus, our ground truth represents the work that a skilled individual would make without any potential for influence or learning effects which might affect subsequent evaluations of the same images.

### Statistical analysis

We compared manual and algorithmic components of: 1. vessel segmentation using Matthew’s Correlation Coefficient (MCC) [24], 2. vessel unaliasing using Mean Absolute Error (MAE) [25], 3. vessel identification using MCC, 4. CBF calculation using Intraclass Correlation Coefficient (ICC) [26].

- **1. Vessel Segmentation** We evaluated the algorithm on whether or not it selected the manually selected voxels (true or false) and whether or not the voxel is a vessel (positive and negative) using the MCC to compare the segmentation of the ICAs and VAs. We restricted the MCC calculation to a region double the width and height of each vessel thereby enabling us to evaluate the MCC for each vessel we processed.
- **2. Vessel Unaliasing** We unaliased 9 phase images obtained from a single adult participant at low VENC (30 cm/s). Unaliasing was performed by each image analyst and the algorithm. We compared the results to paired images acquired at high VENC (60 cm/s), which had no aliasing and thus served as our ground truth. We calculated the MAEs as the mean difference of the unaliased versus ground truth images, and report MAEs for the ICAs and VAs processed by the primary analyst, secondary analyst, and the algorithm.
- **3. Vessel Identification** We wanted to isolate the performance of algorithm vessel identification (and not the prior segmentation). Thus, we used manually segmented images from the primary analyst’s first observation and their resulting identification results as ground truth. We graded the primary analyst (second observation), secondary analyst, and the algorithm on their ability to identify the correct vessel (true or false) and if the vessel was present in the manual segmentation (positive and negative). Because all vessels were visible in the segmentation, the distribution of values were all ‘true positive’ or ‘false negative’. We report the sensitivity for both ICAs and VAs.
- **4. CBF Calculation** Both image analysts and the algorithm calculated CBF using the segmented, unaliased, and identified vessels from the previous components: 1. vessel segmentation, 2. vessel unaliasing, and 3. vessel identification. We calculated the ICC for CBF calculations between the primary analyst (first observation, ground truth) versus primary analyst’s second observation and algorithm. For pediatric participants with neurological tumors, we created a matched (age and sex) cohort without tumors. We calculated the ICC for CBF calculations in the 2 groups between the primary analyst (first observation) versus secondary analyst and algorithm.

### Sample size

We wanted to ensure we used adequate samples to avoid incorrectly reporting equivalent performance. We used a subgroup in the power analyses to estimate effect sizes and standard deviations for each component. We set type I and II errors (α = 0.05, power = 80%), and calculated the number of images needed to evaluate each component. Then, we found the evaluation requiring the most data (3. Vessel Identification, N = 49 images) and exceeded that number of images for all 3 components. For the one component with an objective ground truth (vessel unaliasing) we collected that objective ground truth (unaliased acquisition at an appropriate VENC) and report our values relative to it.

## Results

A total of N = 60 images were collected from N = 59 pediatric participants 3.43 ± 2.60 (range 0.00—12.57) years old and a total of N = 39 images were collected from N = 39 adult participants 57.28 ± 3.87 (range 50.00—64.00) years old. Detailed participant demographics and clinical information are reported in summary Table 1. To acquire a ground truth for the unaliasing component, 9 pairs of images (N = 18 total) were acquired at low and high VENC (30 and 60 cm/s, with and without aliasing) in 1 non-diabetic female adult (age: 29 years old, ethnicity: Latino, race: other). The algorithm performance is reported in summary Table 2, and in detail in sections below.

**Table 1:** Participant Demographic and Clinical Information

Age Range	Demographic and Clinical Information	Characteristic	Number of participants
Pediatric	Sex	Female Male	23 36
	Race	Asian Black Native Hawaiian or other Pacific Islander White More than one race Other Unknown	3 2 1 10 5 23 15
	Ethnicity	Latino Non-Latino Unknown	26 16 17
	Diagnosis	Seizures Non-Neurological Neurological Tumors^1^ Hydrocephalus Optic Nerve Hypoplasia Other^2^	18 15 7 2 2 15
Adult	Sex	Female Male	25 14
	Race	White More than one race Other Unknown	9 1 11 18
	Ethnicity	Latino Unknown	37 2
	Diabetic Status	Non-Diabetic Pre-Diabetic Diabetic Unknown	18 10 9 2
	Comorbidities	High Cholesterol Hypertension Arthritis High Blood Pressure Sleep Disorder Heart Valve Replacement or Repair Traumatic Brain Injury Urinary Incontinence Tremor Other^3^	23 16 15 12 10 5 3 3 2 6

^1^Neurological tumors include 2 volunteers with brain tumors, 1 participant with a brain stem tumor, 1 participant with a central nervous system tumor, and 3 volunteers with neurofibromatosis. ^2^Other diagnoses include 1 participant with each of the following: ataxia, brain mass, cerebellar tonsillar ectopia, cerebral palsy, cerebrovascular aneurysm, encephalitis, headache, hypoxic-ischemic encephalopathy, intraventricular hemorrhage, microcephaly, optic neuritis, sensorineural hearing loss, spinal column mass, ventriculomegaly, and white matter hypoplasia. ^3^Other comorbidities include 1 participant with each of the following: atrial fibrillation, angioplasty, bowel incontinence, seizure, stroke, thyroid disease

**Table 2:** Summary of Component Performance

Processing Component and Performance Metric	Vessel	Comparison (Analyst, Algorithm)	Performance Results	
			**Pediatric**	**Adult**
Vessel Segmentation Mean Matthew’s Correlation Coefficient (MCC)	ICA	primary vs primary	0.918	0.930
	ICA	primary vs secondary	0.861	0.890
	ICA	primary vs algorithm	0.835	0.849
	VA	primary vs primary	0.889	0.914
	VA	primary vs secondary	0.830	0.873
	VA	primary vs algorithm	0.777	0.785
Vessel Unaliasing Mean Absolute Error [ml/min] (MAE)	ICA	primary vs ground truth	NA	0.555
	ICA	secondary vs ground truth	NA	0.555
	ICA	algorithm vs ground truth	NA	0.538
	VA	primary vs ground truth	NA	0.414
	VA	secondary vs ground truth	NA	0.410
	VA	algorithm vs ground truth	NA	0.355
Vessel Identification Sensitivity	ICA	primary vs primary	1.000	1.000
	ICA	primary vs secondary	1.000	1.000
	ICA	primary vs algorithm	0.983	1.000
	VA	primary vs primary	1.000	1.000
	VA	primary vs secondary	1.000	1.000
	VA	primary vs algorithm	0.983	1.000
Total Cerebral Blood Flow Intraclass Correlation Coefficient (ICC)	all	primary vs primary	0.978	0.957
	all	primary vs secondary	0.919	0.810
	all	primary vs algorithm	0.817	0.748

For vessel unaliasing, we were able to acquire an external ground truth. For other components, we treated the first observation from the primary analyst as ground truth. Abbreviations: internal carotid artery (ICA), not applicable (NA), vertebral artery (VA).

1. Vessel Segmentation

The primary image analyst’s first evaluation was used as ground truth. Their second evaluation results were comparable for both ICAs (MCCs = 0.918—0.930, Table 2) and VAs (MCCs = 0.889—0.914, Table 2). The secondary image analyst’s performance versus ground truth was slightly lower for both ICAs (MCCs = 0.861—0.890, Table 2) and VAs (MCCs = 0.830—0.873, Table 2). Similarly, the algorithm was slightly less comparable for both ICAs (MCCs = 0.835—0.849, Table 2) and VAs (MCCs = 0.777—0.785, Table 2). Discrepancies in segmentation tend to happen at the vessel edge, where velocities are low, thus minimizing the impact of different segmentations.

**Fig. 2:**
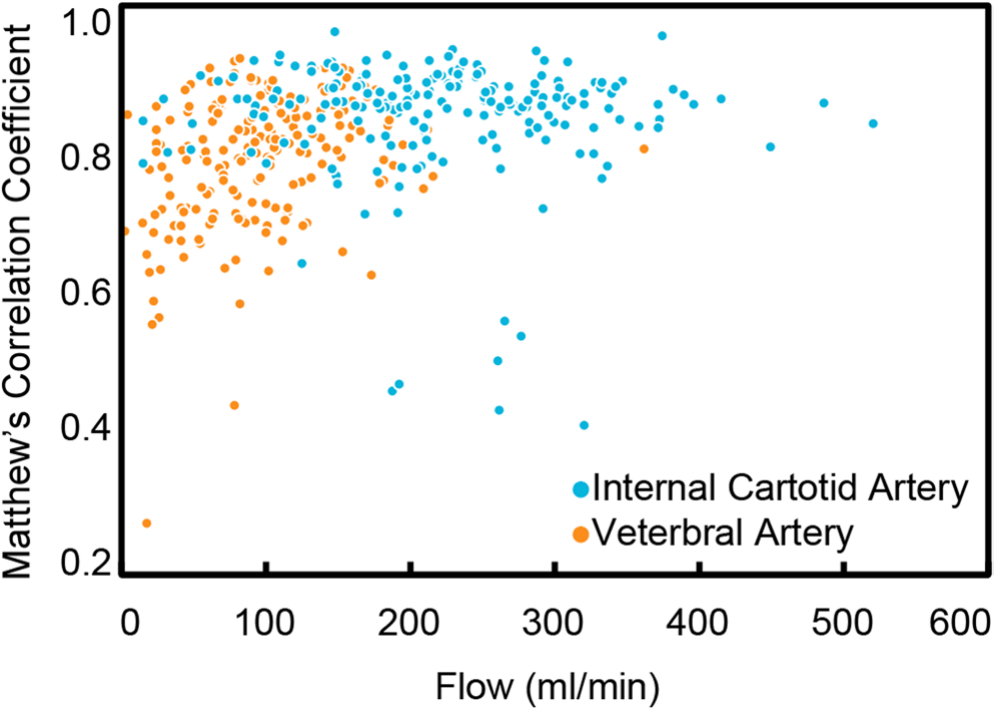
Segmentation comparisons between the primary image analyst and the algorithm show higher Matthew’s Correlation Coefficients (MCCs) in the internal carotid arteries (ICAs) versus vertebral arteries (VAs). The ICA MCCs are higher and more consistent across the flow range. In contrast, the MCCs in VA segmentation are lower and more variable. VAs are generally smaller than ICAs, have lower flows, and are less likely to be perpendicular to the imaging plane, all of which makes them more difficult to segment

The MCCs for individual ICAs and VAs show better performance in ICAs (larger vessels with higher flows) versus VAs (smaller vessels, lower flows) **(**Fig. 2**)**. A two-way Analysis of Variance (ANOVA) of MCCs from the analyst comparisons (primary analyst versus secondary and primary analyst versus algorithm), and 2 different vessels (ICAs and VAs) indicated differences between vessels (p < 0.001), comparisons (p < 0.001), and a vessel-comparison interaction (p = 0.002).

2. Vessel Unaliasing

**Fig. 3:**
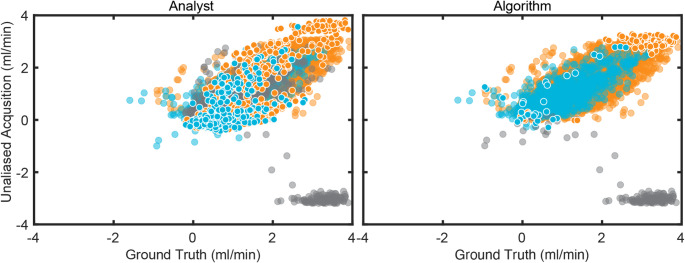
We show the unaliasing performance of the analyst (left), and algorithm (right) as evaluated on all N = 9 pairs of images. These images were collected with parameters to deliberately create a few aliased voxels, which are seen in the lower right corner of each panel. The colors indicate the unaliased results for voxels in the internal carotid artery (ICA, orange) and vertebral arteries (VA, blue). The background of each panel shows unaltered ICAs and VAs, plus the voxels selected for unaliasing (grey). The foreground (outlined) of each panel shows the flows of the grey voxels after unaliasing. The analyst had higher error because they unaliased many voxels that did not require unaliasing, and their manual approach yields more extreme values. In contrast the algorithm selected very few voxels, and the automated approach yielded more moderate values

Data collected without aliasing was used as ground truth. Algorithmic unaliasing had lower error than manual unaliasing for both the ICAs (MAE algorithm = 0.538, analysts = 0.555, Table 2) and VAs (MAE algorithm = 0.355, analysts = 0.410—0.414, Table 2). A two-way ANOVA of MAEs from the comparisons (primary, secondary, and algorithm all versus ground truth), and 2 different vessels (ICAs, VAs) indicated differences between vessels (p < 0.001), but no differences between comparisons (p = 0.639) or vessel-comparison interaction (p = 0.878). Unaliasing performed by the primary image analyst and the algorithm versus ground truth are displayed (Fig. 3).

3. Vessel Identification

**Fig. 4:**
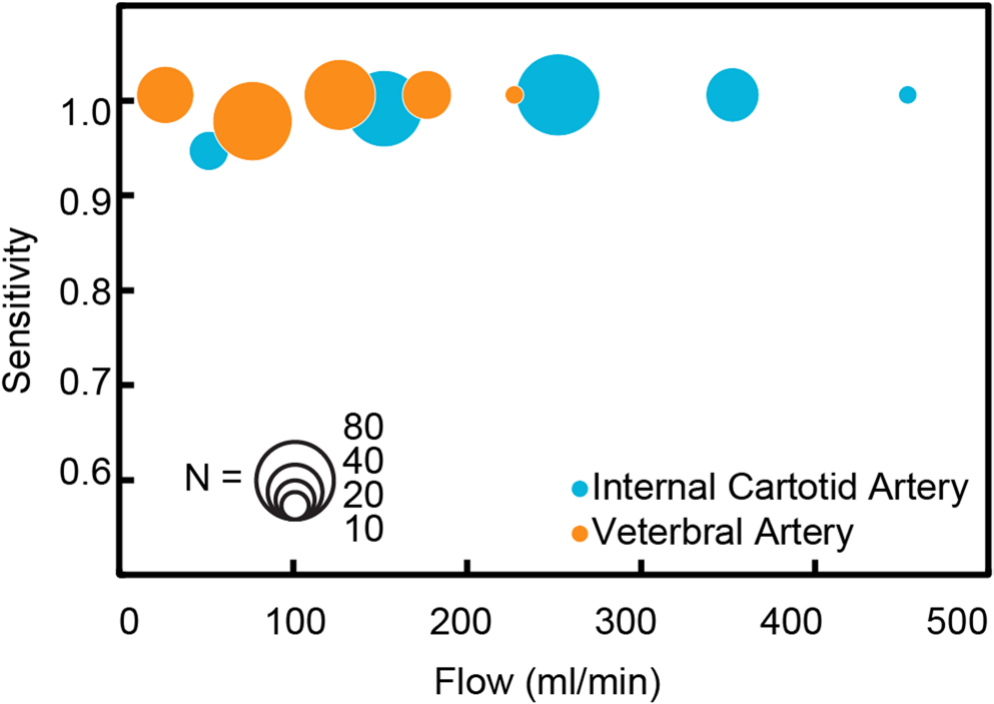
For vessel identification, true positives and false negatives were used to calculate sensitivity across the vessel flow ranges, across all pediatric and adult participants. Results show overall high sensitivity for both ICAs and VAs. At the highest flows, only a few vessels were processed and the results included errors that caused low sensitivity

The primary image analyst’s first evaluation was used as ground truth. Identification by both the primary and secondary analysts were perfect for all images (Sensitivity = 1.000 for all ICAs and VAs, Table 2). Identification by the algorithm was very similar in pediatric (Sensitivity ICAs = 0.983, VAs = 0.983, Table 2) and adult images (Sensitivity ICAs = 1.000, VAs = 1.000, Table 2). False negative errors in the pediatric images included VAs misidentified as ICAs and missed ICAs. Sensitivities for overall ICA and VA identification were 0.990 and 0.990, respectively. Vessel identification was highly sensitive across a range of vessel flows (Fig. 4).

4. CBF Calculation

The primary image analyst’s first evaluation was used as ground truth. CBF measured by the image analysts and algorithm were highly correlated (children: ICCs = 0.817—0.978, adults: ICCs = 0.748—0.957, Table 2). Average CBFs from primary analyst, secondary analyst, and algorithm in children and adults were 524 ± 253 and 682 ± 127 ml/min, respectively (Table 3). The wide variation in pediatric flows is expected due to the wide range of ages we studied. A two-way ANOVA of total CBF from the 4 analyses (ground truth, primary analyst, secondary analyst, and algorithm), and 2 different populations (pediatric, adult) indicated the expected differences between age groups (p < 0.001) and differences between analysts and algorithm (p = 0.016), but no analyst-by-age interaction (p = 0.992) (Table 3). Post-hoc comparisons for all pairs of analyses using the Tukey–Kramer HSD test indicated that only the ground truth (primary analyst’s first evaluation) and algorithm differed (p = 0.033), and all other pairwise comparisons were not significant (p > 0.050).The ICCs indicated slightly higher reliability between algorithm and analyst in children, versus an opposite trend of higher reliability between analysts in adults (Table 2). We report lower reliability in algorithm-analyst comparisons and analyst-analyst comparisons for pediatric participants with tumors (ICC = 0.162 and 0.809) versus comparisons for the matched cohort without tumors (ICC = 0.942 and 0.897).

**Table 3:** Total and Normalized Cerebral Blood Flow Measurements

Analysis	Total Cerebral Blood Flow [ml/min]		Normalized Cerebral Blood Flow [ml/min/100 g]	
	**Pediatric**	**Adult**	**Pediatric**	**Adult**
primary (first observation)	572 ± 260	728 ± 128	55 ± 17	68 ± 14
primary (second observation)	542 ± 267	697 ± 122	52 ± 18	65 ± 14
secondary	494 ± 246	663 ± 109	48 ± 17	62 ± 12
algorithm	487 ± 228	637 ± 130	47 ± 16	59 ± 14
mean	524 ± 253	682 ± 127	50 ± 17	64 ± 14
**Two-Way Analysis of Variance (ANOVA)**				
age group analyses age-analyses interaction	p < 0.001 p = 0.016 p = 0.992		p < 0.001 p = 0.001 p = 0.982	

5. CBF Normalization

**Fig. 5:**
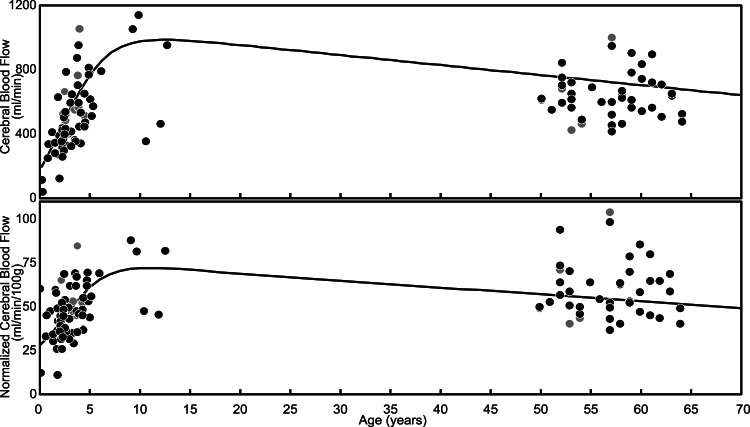
Total Cerebral blood flow (CBF) values (top) calculated by the algorithm and the normalized CBF values (bottom) are displayed across the age range for all participants. Black circles indicate data without any errors or missing data, whereas gray circles indicate data with at least one error in their processing (segmentation, unaliasing, identification, brain weight imputation), and the dark line is a trendline (but not a formal mathematical model) of CBF. We lack data in the 15–50 year age range which we would need to develop such a model

Both total and normalized CBFs are plotted versus age **(**Fig. 5**)**. Most pediatric brain volumes (82%, N = 49) and all adult brain volumes (100%, N = 39) were processed with Free Surfer. Some children’s images failed Free Surfer analysis and were either processed with CAT12 (10%, N = 6), omitted entirely due to severe hydrocephalus (2%, N = 1), or imputed because they lacked any T1 brain image (7%, N = 4) [19].

Mean normalized CBFs from analysts and algorithm in children and adults were 50 ± 17 and 64 ± 14 ml/min/100 g, respectively (Table 3). A two-way ANOVA was also performed on normalized CBF from the 4 analyses (ground truth, primary analyst, secondary analyst, and algorithm), and 2 different populations (pediatric and adult). The results indicated the expected differences between age groups (p < 0.001) and differences between analysis approaches (p = 0.001), but no analyst-by-age interaction (p = 0.982) (Table 3). Post-hoc comparisons for all pairs of analyses using the Tukey–Kramer HSD test indicated that only the ground truth differed versus algorithm (p = 0.005, mean difference = 8.165) and versus secondary analyst (p = 0.022, mean difference = 6.986), and all other pairwise comparisons were not significant (p > 0.050).

## Discussion

We developed an algorithm that automated PC image processing with measures for each key analysis step and had image analysts manually process the same images in a blinded fashion to automated output. Because pediatric PC images are generally of lower quality and more challenging to analyze than adult PC images [12], we evaluated the algorithm on both pediatric and adult PC images. Overall manual and algorithm performance were similar in pediatric and adult images, with better overall algorithm reliability in pediatric images.

Manual processing of MR images is prone to both inter- and intra-operator variability [27], indicating a need for automated approaches with greater consistency. The automated algorithm and manual processing performed similarly for vessel segmentation for both pediatric and adult images. As expected, segmentation results from two analysts were less precise than one analyst performing segmentation twice, and as expected, both were inferior to automation which produces identical results. Automated vessel unaliasing was superior to the manual approach that introduced errors from overestimation and incorrect voxel selection. The algorithm and manual processing also performed similarly for vessel identification for both pediatric and adult images. Overall CBF calculations had high reliability across the automated and manual methods. However, when calculating CBF from ICA and VA flows, the user should check for errors in segmentation, unaliasing, identification, and provide manual correction as needed. For lower quality PC images, ICAs and VAs are harder to segment for both image analysts and the algorithm. Differences of just a few voxels in segmentation can cause errors in unaliasing or flow calculation, leading to incorrectly identified vessels.

All methods had lower reliability in CBF calculations for pediatric participants with neurological tumors than their matched cohort. Images from children with neurological tumors may be more difficult to process, highlighting the need for a precise automated method.

ASL image acquisitions were improved by adopting standardized guidelines [11]. We can also improve PC image acquisitions by creating similar standardized guidelines. PC images vary as differing protocols are implemented by MR technologists at time of acquisition. Efforts to estimate and minimize variations from differing MR vendors, field strengths, gradient strength, gradient slew rate, or coils would improve the reliability of CBF measures between sites and across time at a single site.

The successful development and validation of an automated algorithm for processing of PC images has important implications for measuring CBF in research and eventual clinical use. Algorithmic CBF measures provide potential for increased consistency over time, avoiding human bias in CBF estimation from separate individuals performing manual analysis. Longitudinal studies and those with multiple data acquisition sites would benefit especially in this regard. Acquisition times for PC images are short (less than 2 min) and, when combined with our automated processing algorithm, can be used to validate CBF measures obtained using other methods (e.g. ASL).

### Limitations

Our participants were from 2 different cohorts consisting of young children and adults. We therefore did not evaluate algorithm performance in adolescents. However, we expect the performance of our algorithm to improve in adolescent populations because PC acquisitions are more challenging in young children due to their smaller arteries, lower blood flow, and increased likelihood of movement while scanning. Additionally, some children were imaged under anesthesia, which may lower CBF. Since PC imaging involves calculation of blood flow via voxel sampling of blood velocity within vessel lumens, lower CBF in anesthetized children diminishes image quality. As a result, our pediatric PC images are generally of lower quality compared to those of adults. We included only a small number of children with tumors, and they did not receive ASL imaging, however the main focus of our work was improving PC processing.

The 2 cohorts were imaged on different MR scanners and with different imaging parameters. However, the area of focus for our study was the processing performance on pediatric and adult images. Because scanner and cohort ages were perfectly confounded, we are not able to distinguish differences in processing pediatric and adult images from differences in processing images from different scanners. We cannot rule out that our algorithm’s performance may differ by either scanner acquisition or age. Nevertheless, these differences in image acquisition parameters and quality are useful for validating the robustness of the automated algorithm. If the images were of poor image quality because they did not consider age-dependent factors, then acquisitions with higher image quality would be expected to yield noninferior or superior algorithm performance.

In addition, incorrect placement of the imaging slice may result in capturing oblique ICAs and VAs or external carotid arteries that would complicate segmentation or identification, respectively. For these suboptimal cases, the algorithm has the capability for manual correction in segmentation and identification. Mathematical models can also be employed to accurately impute CBF [28]. Not all scanners may provide the 3 images required by the algorithm from Philips and Siemens scanners (magnitude, complex difference, phase). For components such as vessel segmentation, the algorithm could be developed further to explore the use of only magnitude and phase images.

Common practice involves acquiring dynamic PC images throughout the cardiac cycle, which enables measurement of flow within the cardiac cycle. However, the PC sequence we analyzed acquired and averaged images, and thus it can only yield the average flow within the cardiac cycle. Nevertheless, the algorithm can also be used to calculate flows for dynamic PC images which need the same components, i.e. generate vessel regions of interest, identify vessels, unalias the vessels, and calculate total CBF. An automated approach to analysis (such as ours) is more important when the number of images increases, such as the case with dynamic PC images. Measures from additional images can be fed back into validation of the algorithm and potentially improve components. The weighting parameters in our algorithm were optimized for our images and can be refined further for other data sets.

We calculated flows only for ICA and VA vessels. Nevertheless, using a gated PC sequence and a lower VENC one could measure blood flow through the deformable internal jugular veins to obtain additional information about CBF on the outflow, thereby evaluating the Monroe-Kelly hypothesis. Alternatively, one could measure flow of other fluids such as cerebrospinal fluid through either the cerebral aqueduct or artificial structures, such as the lumen of a ventriculoperitoneal shunt [29].

We evaluated the performance of 2 image analysts, treating the primary analyst as ground truth when needed. Nevertheless, our objective was an automated alternative that has a similar accuracy to manual analysis yet yields high-precision results. Thus, we can eliminate both inter- and intra-observer variability by replacing human analysts with the algorithm. We did not seek to create yet another demonstration of observer variations in medical image processing, which are well-established.

We did not record the processing time for either the analysts or the algorithm, thus we cannot report the potential time savings of the algorithm. Qualitatively, both analysts noted that generating vessel regions of interest and unaliasing vessels were far more time consuming than identifying vessels and calculating total cerebral blood flow. Both analysts also report that manually processing was fatiguing, requiring them to slow their pace to maintain their quality. These observations suggest that the algorithm might be useful as a clinical support tool for radiologists seeking methods that reduce cognitive burden. Implementing such a tool in clinical practice would require integration with the radiologist workstation software, and consideration for their overall workflow.

## Conclusion

We present an efficient algorithm for processing phase contrast images and calculating cerebral arterial flows. The algorithm produces reliable measures of cerebral arterial flows for pediatric and adult images when compared to those generated from manual processing by an image analyst. PC is fast to acquire, relatively insensitive to modeling differences, and radiation free. In contrast, other common CBF measurements used in children are either affected by transit-time (ASL), or involve radiation (PET). Thus, despite the fact that ASL (vs PC) is far more common in research neuroimaging protocols (> 97% vs < 22% of studies, respectively), PC is potentially more suitable for inclusion in routine neuroimaging protocols [18]. The acquisition time and reduced processing time required for phase contrast imaging will together help provide full clinical and research potential for producing high-precision CBF measurements in pediatrics.

## Data Availability

Data and code can be made available upon request to the primary author, requests are subject to institutional policies and procedures.
